# Supplementation with Fish Oil and Genistein, Individually or in Combination, Protects Bone against the Adverse Effects of Methotrexate Chemotherapy in Rats

**DOI:** 10.1371/journal.pone.0071592

**Published:** 2013-08-12

**Authors:** Rethi Raghu Nadhanan, Jayne Skinner, Rosa Chung, Yu-Wen Su, Peter R. Howe, Cory J. Xian

**Affiliations:** 1 Sansom Institute for Health Research, School of Pharmacy and Medical Sciences, University of South Australia, Adelaide, South Australia, Australia; 2 Clinical Nutrition Research Centre, University of Newcastle, Callaghan, New South Wales, Australia; Georgia Regents University, United States of America

## Abstract

Cancer chemotherapy has been shown to induce long-term skeletal side effects such as osteoporosis and fractures; however, there are no preventative treatments. This study investigated the damaging effects of anti-metabolite methotrexate (MTX) subcutaneous injections (0.75 mg/kg BW) for five days and the potential protective benefits of daily oral gavage of fish oil at 0.5 mL/100 g BW (containing 375 mg of n-3 PUFA/100 g BW), genistein (2 mg/100 g BW), or their combination in young adult rats. MTX treatment alone significantly reduced primary spongiosa height and secondary spongiosa trabecular bone volume. Bone marrow stromal cells from the treated rats showed a significant reduction in osteogenic differentiation but an increase in adipogenesis *ex vivo*. Consistently, stromal cells had significantly higher mRNA levels of adipogenesis-related proliferator activator activated receptor-γ (PPAR-γ) and fatty acid binding protein (FABP4). MTX significantly increased the numbers of bone-resorbing osteoclasts and marrow osteoclast precursor cell pool while significantly enhancing the mRNA expression of receptor activator for nuclear factor kappa B ligand (RANKL), the RANKL/osteoprotegerin (OPG) ratio, interleukin-6 (IL-6), and tumor necrosis factor-α (TNF-α) in the bone. Supplementary treatment with fish oil and/or genistein significantly preserved trabecular bone volume and osteogenesis but suppressed MTX-induced adipogenesis and increases in osteoclast numbers and pro-osteoclastogenic cytokine expression. Thus, Fish oil and/or genistein supplementation during MTX treatment enabled not only preservation of osteogenic differentiation, osteoblast number and bone volume, but also prevention of MTX treatment-induced increases in bone marrow adiposity, osteoclastogenic cytokine expression and osteoclast formation, and thus bone loss.

## Introduction

Both clinical and animal studies have reported that chemotherapy causes adverse effects on bone, negatively impacting on bone remodeling and bone mass [1], [2], [3], [4], [5], [6], [7]. Anti-metabolite methotrexate (MTX) is a widely used chemotherapeutic agent in treatment for acute lymphoblastic leukaemia (ALL), non-Hodgkin’s lymphoma, and at lower doses for rheumatoid arthritis and psoriatic arthritis [8]. It competes for the folate binding site of the enzyme dihydrofolate reductase (DHFR), thus disrupting reduction of folic acid to tetrahydrofolic acid responsible for DNA synthesis and cell replication [9], [10]. In treatment of childhood leukaemia, MTX has been shown to cause bone pain, osteopenia and fractures [11], [12]. Previous studies using rat models have demonstrated that MTX decreases trabecular bone volume, which is associated with increased adipogenesis, enhanced osteoclastogenesis, and decreased osteogenesis potential within the bone marrow, and thus a lower osteoblast number but a higher osteoclast density on the bone surface as well as a higher adipocyte density in the bone marrow [1], [3], [13], [14], [15]. Despite these recent findings, the underlying mechanisms for MTX chemotherapy-induced bone loss and marrow require further investigations.

In addition, due to the increasing usage of anti-cancer drugs among cancer patients, it is important to explore potential supplementary treatments which might be useful in protecting bone during cancer chemotherapy. Currently, there is a lack of safe and cost effective treatments against chemotherapy-induced bone loss. The available anti-resorptive therapies using bisphosphonates are known to reduce resorption, increase bone mass and thus have some efficacy in preventing/reducing osteoporosis [16]. However, high costs involved in their administration and also the tendency of forming brittle bones after a long-term usage has been questioned lately [17], [18]. Thus, in the search for supplementary treatments which are safe and non-toxic to protect the bone during cancer chemotherapy, cancer sufferers are increasingly turning to alternative treatments including natural products (nutraceuticals) for better bone health and improved life quality.

Population studies have shown that women consuming high levels of soy products rich in isoflavone genistein and fish rich in omega-3 polyunsaturated fatty acids (n-3 PUFA) have increased bone mass and a lower risk of post-menopausal osteoporosis [19], [20]. The n-3 PUFA eicosapentaenoic acid (EPA) and docosahexaenoic acid (DHA) abundant in fatty fish such as salmon, menhaden and tuna or in their oils are known to have significant anti-inflammatory properties and positive effects on bone metabolism, possibly via suppressing pro-inflammatory mediators like prostaglandin E2 (PGE2), IL-1, IL-6 and TNF-α, which are known to promote osteoclastogenesis and increase bone loss [21], [22]. Both EPA and DHA have been shown to promote bone specific alkaline phosphatase activity, osteoblastogenesis and bone formation and suppress osteoclastogenesis and bone resorption [23], [24], [25], [26], and thus increase bone density in older adults and postmenopausal women [26], [27], [28].

Genistein, a phytoestrogen abundant in soybeans, tofu, tempeh and soymilk, has been shown to have pharmacological properties beneficial for human health including skeletal health [29]. Epidemiological studies have established that the Asian diet with a high level of genistein leads to reduced rates of post-menopausal osteoporosis [30], [31]. Overall, genistein has been shown to anabolically modulate bone cells and benefit bone by stimulating protein synthesis, alkaline phosphatase release, differentiation of osteoblasts [32], [33], production of OPG (an osteoclastogenesis inhibitor) by osteoblasts and bone formation [34], [35]. Genistein has also been shown to suppress the activation of protein phosphatases and nuclear factor-kappa B (NF-kappa B) and Akt signaling pathways, which are known to maintain a homeostatic balance between cell survival and apoptosis, to inhibit osteoclast formation, induce their apoptosis and to suppress bone resorption [32], [33]. Some previous reports have indicated that consumption of genistein benefited bone health while not causing significant adverse effects on breast and uterus tissues [36], [37], [38].

However, it is unknown whether fish oil or genistein has any efficacy in reducing chemotherapy-induced bone defects. Fish oil and genistein individually have anti-inflammatory, anti-osteoclastogenic, pro-osteogenic, and anti-oxidant properties [24], [29], [38], [39], [40], [41]. However, it remains to be investigated whether their combination use may further enhance these beneficial effects on bone health and prevent bone loss caused by chemotherapy. Using a rat model, the current study investigated the damaging effects in osteogenesis, osteoclastogenesis and adipogenesis in long bones caused by MTX treatment and examined the protective effects and potential action mechanisms of supplementary treatments with fish oil and genistein (either individually or in combination).

## Materials and Methods

### Animal Trial and Specimens

This study was approved by the Animal Ethics Committee of SA Pathology/Central Northern Adelaide Health Service of South Australia. Male Sprague-Dawley rats of approximately 6 weeks of age were randomly allocated to eight groups receiving saline (Sal) or MTX injections and oral gavage treatments with water (H_2_O), fish oil (FO), genistein (Gen), or fish oil and genistein in combination (FO+Gen). These 8 groups were: Normal control (Sal+H_2_O), fish oil alone (Sal+FO), genistein alone (Sal+Gen), FO+Gen alone (Sal+FO+Gen), MTX alone (MTX+H_2_O), MTX+FO, MTX+Gen, and MTX+FO+Gen.

For one week prior to MTX or saline injection, rats were pretreated by daily oral gavage with water at 0.5 mL/100 g BW, fish oil alone at 0.5 mL/100 g BW (containing 375 mg of n-3 PUFA/100 g BW), genistein (2 mg/100 g BW) or a combination of fish oil and genistein. Rats were then subcutaneously injected once daily with saline or MTX at 0.75 mg/kg (dosage similar to clinical therapeutic usage) [1], [3] for 5 consecutive days (mimicking the intensive induction phase of treatment for childhood acute lymphoblastic leukaemia (ALL) [42]. Oral gavage treatment was given throughout the whole period and ended one day before kill. For specimen collection, rats were humanely killed by CO_2_ overdose on Day 9 (since the first MTX/saline injection) (a key time point shown to have obvious damaging effects by MTX) as described [3]. The fish oil used was ROPUFA® 75-EE (containing 42% EPA and 22% DHA as ethyl esters, and genistein was Bonistein™ (99.6% pure synthetic genistein) (DSM Nutritional Products, Kaiseraugst, Switzerland).

Peripheral blood collected in lithium-heparin tubes was used for collecting plasma which was stored at −80°C. The proximal left tibia was collected, fixed in 10% formalin for 24 h, decalcified in Immunocal (Decal Corp, Tallman, NY) at 4°C, processed and embedded in paraffin wax for collecting sections of 4 µm thick mounted on positively charged SuperFrost Plus ™ glass slides for histological analysis. Metaphyseal bone (0.4 mm) from the right tibia was obtained, snap frozen in liquid nitrogen and were stored at −80°C for gene expression studies. The remaining tibia, femurs, and humerus were collected and used to obtain bone marrow cells by removing both ends of bones, centrifuging at 0.9 RCF in a microfuge at 4°C for 5 mins. The marrow cells (pooled to yield one sample per rat) were purified by Lymphoprep™ density gradient to obtain the bone marrow mononuclear cells (BMMNC), which were washed with phosphate buffered saline (PBS) and resuspended in basal media consisting of basal minimum essential medium (α-MEM) (Sigma, Sydney, NSW, Australia) containing 10% FBS (Invitrogen, Carlsbad, CA), 50 µg/mL Pen/Strep (Invitrogen), 15 mM HEPES (Sigma) and 130 µM L-ascorbate (Sigma) as described [1]. Upon plating out all cell assays (see below), the remaining mononuclear cell suspension was plated into T75 flasks and cultured with basal media at 37°C and 5% CO_2_. Stromal cells (which adhere to the base of the flasks) were maintained for approximately 10 days until 80% confluence, and then collected and frozen at −80°C until RNA extraction for gene expression studies.

### Histomorphometric Analysis of Growth Plate and Metaphysis

To examine the potential treatment effects on bone formation or bone volume, haematoxylin and eosin (H&E)-stained proximal tibial sections were used for morphometric measurements of growth plate total heights, heights of primary spongiosa and bone volume of primary and secondary spongiosa metaphyseal bone using AnalySIS software [2], [3]. Total height of the growth plate and average primary spongiosa zonal height at the metaphysis were measured parallel to the longitudinal axis of the tibial bone. At the secondary spongiosa (1 mm below the primary-secondary spongiosa transitional line), total areas and the areas of all trabecular cores were measured, which were then used to calculate the bone volume to total tissue volume fraction (BV/TV %) as described [1], [2], [3], [4], [14].

### Numbers of Bone Surface Osteoblasts and Osteoclasts and Bone Marrow Adipocytes

Osteoblasts and osteoclasts are cells involved in bone formation and bone resorption respectively; their densities on trabecular bone surface were measured to assess treatment effects in bone. Staining for tartrate-resistant acid phosphatase (TRAP, a marker for osteoclasts) was performed using naphtal AS-BI phosphate (Sigma) and a solution of pararosanilline and sodium nitrate as described [7]. Osteoclasts (TRAP-positive cells containing at least 3 nuclei) along the trabecular surface were counted within primary spongiosa and secondary spongiosa and expressed as osteoclasts per mm^2^ trabecular bone area. At both the primary and secondary spongiosa on H&E stained sections, the osteoblast numbers (mononuclear cuboidal shaped cells lining trabecular surface) were also counted and expressed as total osteoblasts per mm^2^ of trabecular bone area as described [2]. Adipocyte density was determined at the lower secondary spongiosa bone marrow (cells/mm^2^ marrow area) by measuring the average of adipocytes across 3 sequential images as described [15].

### Colony Forming Unit Fibroblast (CFU-f) Assay and Alkaline Phosphatase (ALP) Staining

To determine treatment effects on the size of osteoprogenitor cell pool, a CFU-f assay followed by ALP staining was performed as described [1], [43]. Briefly, bone marrow cells were cultured at 1×10^6^ in 6-well tissue culture dishes with basal media (described above) and were allowed to adhere and proliferate to form colonies for 14 days with media changed twice a week. At the end of the culture, cells were fixed with 10% formalin and stained for ALP activity. Subsequently, plates were stained with toluidine blue (Sigma) as an assessment of total numbers of colony formation. ALP^+^ or toluidine^+^ colonies (containing more than 50 cells in a cluster in each colony) were counted under a light microscope. The numbers of ALP^+^ colonies were expressed as percentage of total CFU-f colonies.

### Mineralization Assay and Alizarin Red Staining

As means to assess the treatment effects on mineralization capacity, bone marrow cells were plated out at a plating density of 2×10^6^ in a T25 filtered flasks with basal media for a week. For initiation of mineralization, basal media was then replaced by mineralization media containing basal media supplemented with 10 nM dexamethasone and 10 mM β–glycerolphosphate (Sigma) and were cultured for an additional 11 days with media refreshed twice per week. Mineralizing colonies was assessed upon alizarin red staining with positive colonies (red nodules) counted, followed by toluidine blue staining and expressed as a percentage of alizarin red^+^ of total CFU-f colonies.

### Ex-vivo Adipogenesis Assay and Nile Red Staining

To assess treatment effects on adipogenesis in the bone marrow, bone marrow stromal cells were obtained as described above and were cultured for 7 days in T25 flasks at a plating density of 2×10^6^ with normal basal media. For adipogenic induction, cells were fed the basal medium supplemented with 1 uM dexamethasone (Sigma), 0.5 mM methyl-isobutylxanthanine (Sigma) and 100 uM indomethacin (Sigma) for another 7 days as described [15], [44]. Nile red staining was used to identify adipogenic colonies under fluorescence microscope (excitation 485 nm, emission 525 nm), followed by toluidine blue staining, and total adipogenic colonies were expressed as percentage of Nile red^+^ of total CFU-f colonies.

### Ex-vivo Osteoclast Formation Assay and TRAP Staining

Treatment effects on osteoclastogenesis were analyzed using an *ex vivo* osteoclast formation assay via the RANKL/M-CSF system [1], [45], [46]. Bone marrow cells obtained from the rats were cultured in α-MEM supplemented with 10% FBS, 50 µg/ml Pen/Strep and 15 mM HEPES. Upon culturing overnight, non-adherent hematopoietic cells were collected and were plated in 96-well trays at a density of 3×10^5^ cells/well in triplicate, and cultured in the above medium plus 10 ng/ml macrophage colony-stimulating factor (M-CSF) (Peprotech, Rocky Hill, NJ). Starting from the following day, 30 ng/ml of RANKL (Peprotech) was added into this M-CSF-containing medium; and cells were maintained for 8 days with medium change once every 3 days. At the end of culture, cells were fixed and stained for TRAP as described [1]. Numbers of osteoclasts formed are presented as TRAP^+^ multinuclear cells/mm^2^.

### Quantitative RT-PCR Analysis of Gene Expression

For analyzing levels of expression of genes related to osteogenic and adipogenic differentiation, total RNA was extracted from bone marrow stromal cells (obtained and cultured as described above) using RNAqueous®-Micro Kit (Ambion, Applied Biosystems, Melbourne, Australia) and treated with DNAse using DNA-*free* kit (Ambion). For examining treatment effects on expression of pro-inflammatory cytokines and osteoclastogenesis regulatory genes in bone, RNA was extracted from the metaphyseal bone samples (obtained as described above) using Tri-reagent (Sigma) [47]. cDNA synthesis from the RNA was done using an High Capacity RNA to cDNA kit (Applied Biosystems). Quantitative PCR assays were run on a 7500 Fast Real-Time PCR System (Applied Biosystems) in duplicate using specific primers (Table 1) [47] (ordered from Geneworks, Adelaide, SA, Australia). Relative expression was calculated using the comparative Ct (2^−Δ^Ct) method, with Cyclophilin A (Cyc A) as the endogenous control.

**Table 1:** List of primers used in RT-PCR.

Gene	Forward Primer (5′-3′)	Reverse Primer (5′-3′)
IL-1	GTTTCCCTCCCTGCCCTCGAC	GACAATGCTGCCTCGTGA
IL-6	CAGCGATGATGCACTGTCAGA	CCAGGTAGAAACGGAACTCCA
IL-4	ACAAGTCTGGGGTTCTCGGTG	CGGTGCAGCTTCTCAGTGAGTTC
IL-10	GGCCATTCCATCCGGGGTGA	GAAATCGATGACAGCGTCGCAGC
TNF-α	ATGGCCCAGACCCTCACACTCAGA	CTCCGCTTGGTGGTTTGCTACGAC
RANK	GGGAAAACGCTGACAGCTAATC	GGTCCCCTGAGGACTCCTTATT
RANKL	CCGTGCAAAGGGAATTACAAC	GAGCCACGAACCTTACATCA
OPG	CACAGCTCGCAAGAGCAAACT	ATATCGCGTTGCACACTGCTT
OCN	ATTCACCACCTTACTGCCCTCCTG	GCTGGCCCTGACTGCATTCTG
CYC-A	GAGCTGTTTGCAGACAAAGTTTC	CCCTGGCACATGAATCCTGG
PPAR-γ	TCCTCCTGTTGACCCAGAGCAT	AGCTGATTCCGAAGTTGGTGG
FABP 4	GGAATTCGATGAAATCACCCC	TGGTCGACTTTCCATCCCACT
Osx	GCTTTTCTGTGGCAAGAGGTTC	CTGATGTTTGCTCAAGTGGTCG
Runx2	TCACAAATCCTCCCCAAGTGG	GAATGCGCCCTAAATCACTGA

### Multiplex Pro-inflammatory and Anti-inflammatory Cytokine Assay

To examine treatment effects on plasma levels of pro-inflammatory and anti-inflammatory cytokines, the Bio-Plex Pro™ magnetic bead-based multiplex rat cytokine assay kit (Biorad, Hercules, CA) was specially customized and used to detect IL-4, IL-10, IL-6, TNF-α and IL-1 in the plasma samples. Briefly, the capture antibody–coupled beads were first incubated with antigen standards or samples (in duplicate) followed by incubation with biotinylated detection antibodies. After washing, the beads were incubated with a reporter streptavidin-phycoerythrin conjugate (SA-PE). Following removal of excess SA-PE, the beads were passed through the Bio-Plex array reader, which measures the fluorescence of the bead and of the bound SA-PE. All washes were performed using a Bio-Plex Pro wash station. Data acquisition was performed using Bio-Plex Manager™ software 5.0 at high PMT setting.

### Statistics

Data are presented as means ± SEM and were analysed by a one-way analysis of variance (ANOVA) using GraphPad Prism 5 (GraphPad Software, San Diego, CA). When significance (P<0.05) was achieved, a *post hoc* analysis of groups was performed using a Tukey's test. Histogram bars with differing letters denote mean values which are significantly different from each other (P<0.05).

## Results

### Treatment Effects on Primary Spongiosa Height and Trabecular Bone Volume

**Figure 1:**
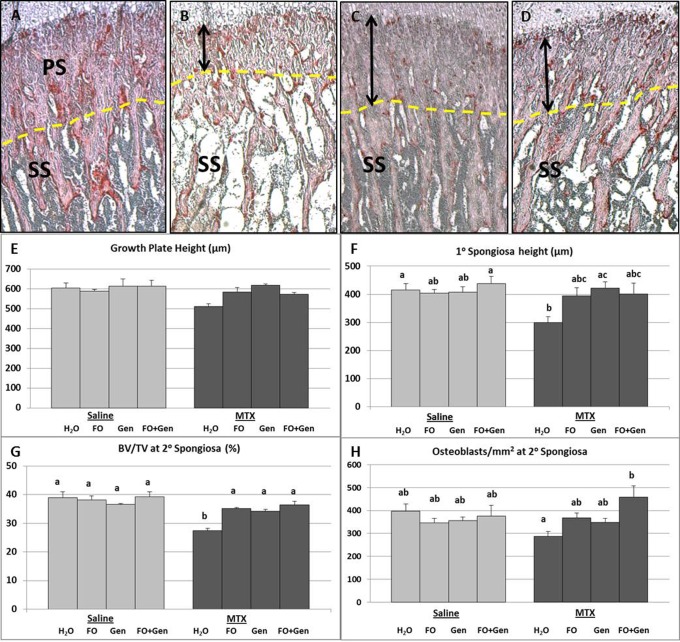
Effects of MTX with or without fish oil (FO) and/or genistein (Gen) supplementation on growth plate, primary and secondary spongiosa. Paraffin sections of the tibial metaphysis region (PS =  Primary spongiosa, SS =  Secondary spongiosa, which are separated by a dashed line) of (**A**) a normal rat, (**B**) a MTX+H_2_O treated rat showing reduced height of primary spongiosa and. metaphyseal bone volume, (**C**) a MTX+FO treated rat, and (**D**) a MTX+Gen treated rat. (**E**) Growth plate total height (µm). (**F**) Primary spongiosa height (µm). (**G**) Secondary spongiosa BV/TV (%). (**H**) Secondary spongiosa osteoblast/mm^2^ trabecular bone area. Values are means ± SEM; n = 7–8 for all groups. Labelled means without a common letter differ (P<0.05).

Histomorphometric measurements revealed no significant changes in the total height of the growth plate across the treatment groups (P>0.05) (**Fig. 1A**). A significant reduction of the primary spongiosa height was noted in the MTX alone group when compared to the control (Sal+H_2_O) group (P<0.05) and Sal+FO+Gen (P<0.01). All supplemented groups (MTX+FO, MTX+Gen, MTX+FO+Gen) were considerably higher than the MTX alone group and were not statistically different from the Sal+H_2_O, although only MTX+Gen group was significantly higher than the MTX alone group (P<0.05) (**Fig. 1A–1D, 1F**). Measurements of the BV/TV (%) within the secondary spongiosa revealed a significant reduction in MTX alone group compared to Sal+H_2_O, Sal+FO, Sal+Gen and Sal+FO+Gen groups (P<0.001) (**Fig. 1A–1D, 1G** **)**. MTX+FO (P<0.01), MTX+Gen (P<0.05) and MTX+FO+Gen (P<0.01) treatments had significantly preserved the bone volume, which was reduced by MTX alone (**Fig. 1G**).

### Treatment Effects on Osteoblast Numbers and Osteogenic Differentiation Potential

**Figure 2:**
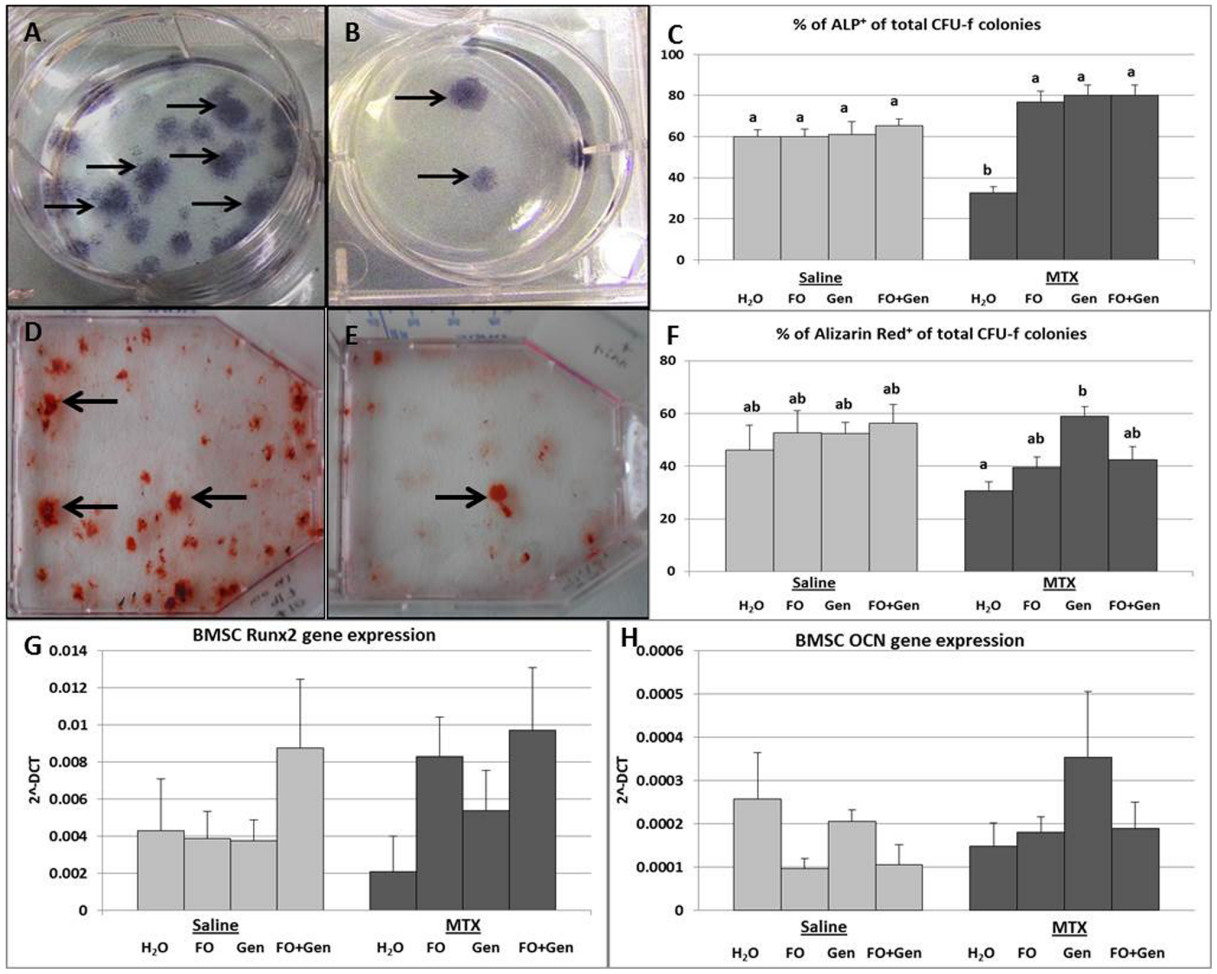
Effects of MTX alone or with supplementary treatment with (FO) and/or genistein (Gen) on osteogenic differentiation potential of bone marrow stromal cells isolated from treated rats. Images of a culture well showing bone marrow stromal cell-derived CFU-f colonies stained positive for alkaline phosphatase (ALP, arrows) of (**A**) a control rat and (**B**) a MTX alone treated rat on day 9 post the first MTX injection. (**C**) Treatment effects on size of osteoprogenitor cell pool in bone marrow. Mineralizing colonies stained positive by Alizarin Red (arrows) of (**D**) a control rat and (**E**) a MTX alone treated rat. (**F**) *Ex vivo* mineralization assay with bone marrow cells isolated from rats. RT-PCR relative mRNA expression of (**G**) Runx2 and of (**H**) bone matrix protein osteocalcin (OCN) assessed in bone marrow stromal cells of treated rats (relative to Cyclophilin-A). Labelled means without a common letter differ (P<0.05).

At the secondary spongiosa, MTX alone treatment caused no significant reductions in osteoblast numbers when compared to all of the control groups, MTX+FO and MTX+Gen groups (P>0.05) (**Fig. 1H**). However, osteoblast density was significantly preserved in the MTX+FO+Gen group compared to the MTX alone group (P<0.05). *Ex vivo* CFU-f assay plus ALP staining revealed a significantly lower percentage of ALP^+^ CFU-f colonies formed by the bone marrow stromal cells from the MTX alone group when compared to Sal+H_2_O, Sal+FO, Sal+Gen (P<0.05) and Sal+FO+Gen (P<0.01) groups (**Fig. 2A–2C**). Compared to the MTX alone group, the supplemented groups (MTX+FO, MTX+Gen and MTX+FO+Gen) had significantly more ALP^+^ CFU-f colonies (P<0.001, P<0.001**,** P<0.01 respectively) (**Fig. 2C**). The ability of CFU-f colonies to mineralize was also determined in this current study (by counting alizarin red-stained colonies) (**Fig. 2D, 2E**). It was revealed that, while MTX alone group did not significantly produce fewer mineralizing colonies when compared to all the saline-treated control groups (P>0.05), only MTX+Gen group had a significantly more mineralizing colonies than MTX alone group (P<0.05) (**Fig. 2F**).

Real time RT-PCR analyses of the mRNA expression levels of osteogenic transcription factors Runx2 and Osx in bone marrow stromal cells levels of Runx2 (**Fig. 2G**) and Osx (data not shown) were not significantly affected in the MTX alone group when compared to all of the other treatment groups (P>0.05). Expression of bone matrix protein OCN in the stromal cells were not significantly affected by the different treatments (P>0.05) (**Fig. 2H**).

### Treatment Effects on Adipocyte Numbers and Adipogenic Differentiation

**Figure 3:**
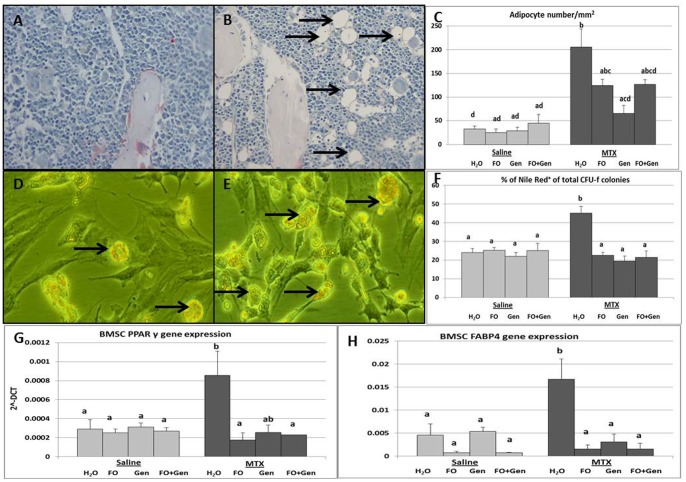
Effects of MTX with or without (FO) and/or genistein (Gen) supplementation on bone marrow adiposity and adipogenesis potential *ex vivo*. H&E-stained sections of tibial lower secondary spongiosa in a (**A**) control rat and (**B**) a MTX alone treated rat. (**C**) Adipocyte numbers on bone histology sections. (**D**) Nile Red-stained images of cultures showing adipocyte formation in an *ex vivo* adipogenesis assay with bone marrow stromal cells of a control rat and (**E**) a MTX+H_2_O treated rat. (**F**) Quantification of Nile Red^+^ colonies in an *ex vivo* adipogenesis assay from bone marrow cells of treated rats. RT-PCR relative gene expression analysis of adipogenesis related genes (**G**) PPARγ and (**H**) FABP4 assessed in the isolated bone marrow stromal cells. Labelled means without a common letter differ (P<0.05).

Histological measurements of adipocyte numbers at the metaphyseal (secondary spongiosa) and diaphyseal junction area revealed a significantly higher adipocyte number in the MTX alone group compared to all of the control groups (P<0.001) (**Fig. 3A–3C**). While fish oil supplementation (MTX+FO) tended to prevent MTX-induced marrow adiposity (P>0.05 vs MTX alone group), the MTX+FO group still had more adipocytes than the Sal+H_2_O group (P<0.05) (**Fig. 3C**). However, genistein supplementation (MTX+Gen) significantly suppressed MTX treatment-induced increased adipocyte numbers (P<0.001 vs the MTX alone group; P>0.05 vs Sal+ H_2_O group).

Consistently, *ex vivo* adipogenic differentiation assessment of the bone marrow stromal cell samples revealed significantly more Nile red^+^ colonies in cultures from the MTX alone group than the control or supplement alone groups (P<0.001) (**Fig. 3D–3F** **)**. All the supplementary treatment groups (MTX+FO, MTX+Gen and MTX+FO+Gen) had significantly fewer Nile red^+^ colonies in comparison to MTX alone group (P<0.001), which had similar levels as the control groups (P>0.05) (**Fig. 3F**).

Consistent with the finding of increased adipocyte formation upon MTX treatment, expression of adipogenesis regulatory genes PPAR-γ and FABP4 in cultured bone marrow stromal cells was significantly elevated in the MTX alone group compared to all of the control groups (P<0.05) (**Fig. 3G, 3H**). All the supplementary groups significantly attenuated the MTX-induced increased expression of FABP4 (P<0.01 vs MTX alone group). MTX+FO and MTX+FO+Gen (P<0.05 and P<0.01 respectively) treatments also significantly suppressed MTX-induced increased expression in PPAR-γ (**Fig. 3G, 3H**).

### Treatment Effects on Osteoclast Density and Formation

**Figure 4:**
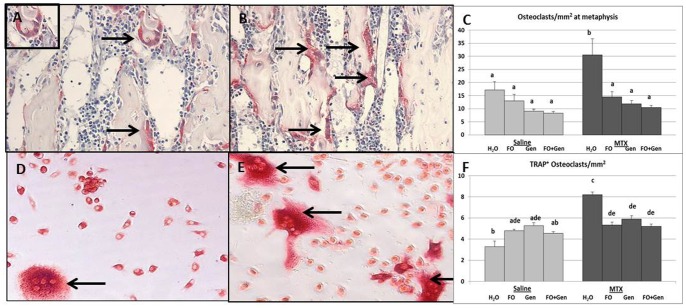
Effects of MTX with or without (FO) and/or genistein (Gen) supplementation on osteoclastogenesis potential. Images of TRAP-stained tibial metaphysis (arrows pointing multinucleated TRAP-positive osteoclasts) of (**A**) a control rat and (**B**) a MTX alone treated rat on day 9 post the first MTX injection showing more osteoclasts present. (**C**) Average osteoclast numbers at tibial primary and secondary spongiosa. Images of TRAP positively-stained cells formed (arrows pointing multinucleated TRAP-positive osteoclast-like cells) in an *ex vivo* osteoclastogenesis assay of (**D**) a control rat and (**E**) a MTX alone treated rat on day 9 post the first MTX injection showing more osteoclasts formed. (**F**) *Ex vivo* osteoclast formation from bone marrow cells isolated from treated rats. Labelled means without a common letter differ (P<0.05).

TRAP staining and histological measurement revealed that, compared to the Sal+H_2_O control and all other control groups, the MTX alone group displayed a significantly increased number of osteoclasts on trabecular surface at the metaphysis (P<0.05) (**Fig. 4A–4C**). However, the MTX-induced increase in osteoclast density was significantly attenuated in all three supplement groups (MTX+FO, MTX+Gen and MTX+FO+Gen, P<0.01, P<0.001, P<0.001, respectively, compared to MTX alone group). Consistent with the histological observations, *ex vivo* osteoclastogenesis assay with isolated bone marrow cells showed that there were significantly more TRAP^+^ multinucleated cells formed from the bone marrow from the MTX alone group than from all control groups (Sal+H_2_O, Sal+FO, Sal+Gen, and Sal+FO+Gen) (P<0.001) (**Fig. 4D–4F**). All supplemented groups (MTX+FO, MTX+Gen and MTX+FO+Gen) had significantly suppressed the osteoclastogenic potential induced by MTX (P<0.001 compared to MTX alone group) (**Fig. 4F**).

### mRNA Expression of Pro-inflammatory Cytokines and Osteoclastogenesis-related Molecules

**Figure 5:**
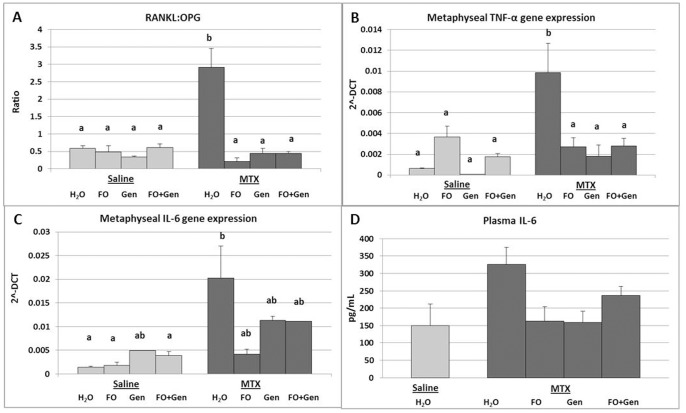
Effects of MTX with or without (FO) and/or genistein (Gen) supplementation on expression of osteoclastogenesis-regulatory or related genes. Levels of mRNA expression in metaphysis bones of treated rats as quantitated by real time RT-PCR: (**A**) RANKL/OPG ratio, (**B**) TNF-α, and (**C**) IL-6. (**D**) Levels (pg/mL) of circulating IL-6 protein in plasma samples of treated rats as measured by multiplex cytokine assay. Labelled means without a common letter differ (P<0.05).

Quantitative RT-PCR expression analyses of pro-inflammatory cytokines and osteoclastogenesis regulatory molecules showed that, consistent with the enhanced osteoclastogenesis and osteoclast density at the metaphysis induced by MTX treatment, MTX alone treated rats had significantly higher mRNA expression of RANKL when compared to rats treated with Sal+H_2_O (P<0.05), Sal+FO (P<0.05) and Sal+Gen (P<0.01) (data not shown). However, mRNA gene expression of OPG was not significantly different between the rats treated with MTX alone and with Sal+ H_2_O (P>0.05). MTX+FO group had a significant upregulation in the OPG levels in comparison to groups treated with MTX+ H_2_O, Sal+FO, Sal+Gen, Sal+FO+Gen (P<0.001 respectively) and MTX+FO+Gen (P<0.05) (data not shown). As a result, there was a significant upregulation in the RANKL/OPG expression ratio in the MTX alone group compared to all the control groups (P<0.001) (**Fig. 5A**). All supplementary treatments (MTX+FO, MTX+Gen and MTX+FO+Gen) significantly attenuated the RANKL/OPG ratio compared to the MTX alone group (P<0.001) **(** **Fig. 5A** **)**. Similarly, expression of TNF-α was significantly upregulated in the MTX alone group compared to all control groups (Sal+H_2_O, Sal+Gen, Sal+FO+Gen, P<0.001; and Sal+FO, P<0.05) (**Fig. 5B**). However, this upregulation was significantly suppressed in all the supplementary treatment groups (MTX+FO, MTX+Gen and MTX+FO+Gen, P<0.001, P<0.001, P<0.01, respectively, compared with MTX alone group) (**Fig. 5B**).

**Figure 6:**
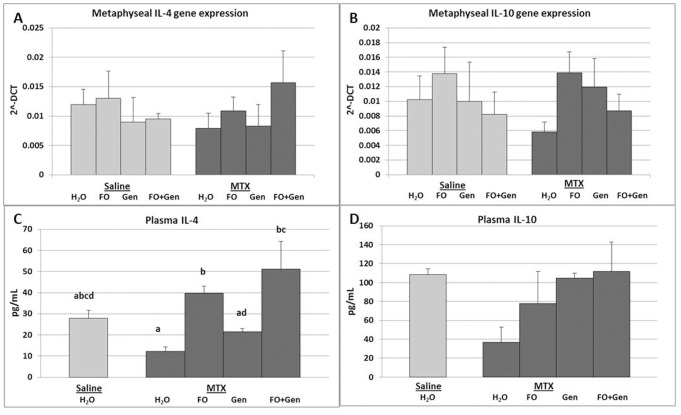
Effects of MTX with or without (FO) and/or genistein (Gen) supplementation on expression of anti-inflammatory cytokines IL-4 and IL-10. Levels of mRNA expression in metaphysis bones of treated rats as quantitated by real time RT-PCR: (**A**) IL-4 and (**B**) IL-10. Protein levels (pg/mL) of circulating (**C**) IL-4 and (**D**) IL-10 in plasma samples of treated rats as measured by multiplex cytokine assay. Labelled means without a common letter differ (P<0.05).

IL-6 gene expression was also significantly induced with MTX alone treatment compared to Sal+H_2_O control (P<0.01) (**Fig. 5C**); however, all supplementary treatments did not significantly counteract MTX-induced increase in IL-6 expression (P>0.05 compared to MTX alone group) (**Fig. 5C**). Expression of IL-1 was shown not to be affected significantly by MTX treatment nor by all the supplementary treatments, although it tended to be elevated in MTX alone group (P>0.05 compared to Sal+H_2_O group) and MTX+Gen and MTX+FO+Gen groups tended to suppress this tendency (P>0.05) (data not shown). Levels of mRNA expression in bone of anti-inflammatory cytokines IL-4 (**Fig. 6A**) and IL-10 (**Fig. 6B**) were also analysed which showed no significant differences across the treatment groups (P>0.05).

### Levels of Pro- or Anti- Inflammatory Cytokines in Plasma Samples

A multiplex cytokine ELISA assay with the plasma samples revealed no significant alterations in the level of pro-inflammatory cytokine IL-6 across the treatment groups (P>0.05) (**Fig. 5D**). Interestingly, while MTX treatment alone did not significantly reduce the plasma anti-inflammatory cytokine IL-4 level (P>0.05), fish oil and/or genistein supplementation in MTX-treated rats reversed this trend to the extent that levels of plasma IL-4 were significantly greater in MTX+FO and MTX+FO+Gen groups than in the MTX alone group (P<0.05) (**Fig. 6C**). Plasma levels of pro-inflammatory cytokine IL-10 were not significantly altered in all of the treatment groups (P>0.05) (**Fig. 6D**). Analyses of other cytokines such as IL-1, TNF-α and RANKL in plasma samples did not reveal obvious differences among treatment groups (P>0.05) (data not shown).

## Discussion

MTX chemotherapy has been shown to cause osteopenia, bone pain and fracture [11], [12], [48], [49], for which the underlying mechanisms remain unclear and there is a lack of preventative therapeutic options. The current study further defined the effects of MTX treatment in rats and also explored the potential protective effects of oral consumption of anti-inflammatory fish oil and anti-oxidant and osteotrophic genistein, either individually or in combination, against MTX-induced bone loss. Consistent with the previous observation [1], [3], [14], the current study revealed that MTX treatment significantly decreased the primary spongiosa height and secondary spongiosa trabecular bone volume. Although the primary spongiosa region of the metaphysis is derived from the growth plate, these observed changes in the metaphysis might be due to the observed significant increase in osteoclast recruitment and resorption. The significantly increased osteoclast numbers and thus resorptive activity while no significant differences in the density of osteoblasts at both primary and secondary spongiosa would have contributed to the significant reduction in the primary spongiosa height and secondary spongiosa bone volume. However, it was clear that supplementation with fish oil and/or genistein had significantly protected the height of the primary spongiosa and preserved the bone volume at the metaphysis which were reduced by the MTX alone treatment. As discussed below, this might be due to the ability of these supplements to not only suppress osteoclastogenesis/osteoclast numbers but also to preserve the bone marrow osteogenesis/bone surface osteoblast numbers.

Since studies have shown that a shift in differentiation of bone marrow mesenchymal stem or stromal cells (MSCs) towards the adipocyte lineage over the osteoblast lineage can ultimately lead to bone loss [15], [50], the current study investigated the treatment effects on the MSC lineage commitment in the bone marrow. Although MTX treatment did not cause any significant reduction in osteoblast numbers on the trabecular surface, it caused a statistically significant suppression in osteogenic differentiation potential as exhibited by ALP^+^ CFU-f and mineralizing assays within the bone marrow stromal cell population. However, no statistically significant reduction was observed in mRNA gene expression of osteogenic transcription factors Runx2 and OCN in bone marrow stromal cells. Supplementary treatment with fish oil and/or genistein however prevented the reduction in osteogenic differentiation induced by MTX as revealed by ALP^+^-CFU-f and mineralizing assays. This was also consistent with the preservation of osteoblast numbers on the trabecular surface. Our findings suggest that fish oil and/or genistein preferentially enhanced the differentiation of the marrow stromal cells down the osteogenic lineage, thus contributing to the preserved bone mass during MTX treatment. Our findings are consistent with the previous findings of osteotrophic activity of n-3 PUFA and genistein in animals or post-menopausal women with estrogen deficiency [25], [51], [52], [53], [54].

Consistent with findings in a previous study [15], while reducing the osteogenic potential, MTX caused an increased marrow adiposity with a greater adipocyte number in the bone marrow. Consistent with the increased adipogenesis after MTX treatment, the current study also exhibited a significant upregulation of adipogenesis-related genes, FABP4 and PPAR-γ, in bone marrow stromal cells isolated from the treated rats. Together, the current and the previous studies [15], [44] indicate that the increased adipogenesis and reduced osteogenesis within the bone marrow of MTX treated rats reflect a selective differentiation of stromal progenitor cells down an adipogenic pathway in the expense of osteogenesis. Consistent with this, a clinical study showed that patients with acute myeloid leukaemia (AML) exhibited increased fat cell formation within the bone marrow immediately after intensive chemotherapy [55]. Furthermore, the current study showed that fish oil and/or genistein supplementation had stimulated osteogenesis while concurrently inhibiting MTX-induced adipogenesis in the bone and bone marrow as revealed by the histological, ex vivo adipogenesis culture and PPAR-γ and FABP4 gene expression studies. Our findings with the MTX chemotherapy setting are consistent with previous observations that these supplements have the ability to suppress expression of adipogenic transcription factor PPAR-γ and adipogenic differentiation of bone marrow stromal cells *in vitro* [56], [57], [58], [59]. Although the underlying action mechanisms remain to be studied, our study has demonstrated the ability of fish oil and/or genistein to promote osteogenesis while attenuating MTX-induced adipogenesis, thus contributing to preserving bone volume and preventing marrow adiposity.

In the current study, a significant increase in the numbers of osteoclast at metaphysis and enhanced osteoclastogenesis in the bone marrow were observed in the MTX alone group, which were consistent with previous findings [1], [46], [60], [61]. In the current study, as mentioned earlier, the osteoblast numbers were only slightly affected in the metaphysis by the MTX treatment; thus the significant increase in osteoclast numbers in the MTX treated group would likely have contributed to the reduced bone volume. This observation was consistent with another study involving prolonged use of low dose MTX, in which a reduced bone density was observed to be associated with an increased osteoclast number [62]. In the current study, supplementary treatment with fish oil and/or genistein was found to significantly prevent MTX treatment-induced increased osteoclast presence on bone surface and osteoclastogenesis in the bone marrow, suggesting that fish oil and/or genistein have an anti-osteoclastic property and may prevent osteoclast formation and bone loss during MTX chemotherapy. Our anti-osteoclastogenesis observation with fish oil and/or genistein with the MTX chemotherapy setting were consistent with anti-osteoclastogenic property of these supplements in estrogen deficiency setting in rats [24], [33].

Osteoclasts differentiate from the monocyte/macrophage cell lineage under the control of M-CSF and RANKL as well as promotion from some pro-inflammatory cytokines [63]. A recent study has demonstrated that MTX treatment can create an inflammatory microenvironment in bone that coincided with increased osteoclast formation and numbers [46]. In addition, an increase in serum levels of IL-8 and TNF-α has been found in patients subjected to chemotherapy drugs like epirubicin, vincristine, cyclophosphamide, etoposide and prednisone [64], [65], [66]. The current study has also examined treatment effects on expression of RANKL, inhibitor OPG and a number of pro-inflammatory cytokines that are known to enhance osteoclast formation, number and activity. It was found that, accompanying a significant increase in osteoclast presence on bone surface and osteoclastogenesis after MTX treatment, significantly increased expression of RANKL but decreased expression in OPG (thus a significantly higher RANKL/OPG ratio) were noted in the MTX treated group, suggesting MTX chemotherapy modulates the key osteoclastogenic signal. In addition, consistent with findings in a recent study [46], mRNA expression of osteoclastogenic cytokines TNF-α and IL-6 within the bone was increased IL-6 protein levels were elevated in the plasma. *In vitro* studies have revealed that TNF-α, IL-1 and IL-6 are capable of inducing active resorption by mature osteoclasts [63], [67]. These data indicate that MTX treatment may create an inflammatory or osteoclastogenic microenvironment in the bone promoting osteoclast formation and activity. Consistent with the ability of fish oil and/or genistein in inhibiting MTX-induced osteoclast formation and bone resorption in this study, these supplements blunted MTX-induced elevated RANKL/OPG ratio and expression of IL-6 and TNF-α. Although the exact mechanism of action of these supplements in suppressing osteoclastogenesis still requires further investigation, rodent and human studies in estrogen deficiency or non-challenged settings have also shown the ability of n-3 PUFAs and genistein to suppress IL-6, IL-1 and TNF-α expression and/or to lower RANKL/OPG ratio, thus attenuating osteoclast formation and activity [29], [32], [40], [54], [68], [69], [70], [71]. Similarly, a clinical study has shown that fish oil supplementation in rheumatoid arthritis patients was associated with decreased production of IL-1 and TNF-α [72]. Therefore, fish oil and genistein in the current study were shown to suppress MTX treatment-induced expression of the pro-inflammatory cytokines and RANKL/OPG ratio, which may have contributed to reduced osteoclast differentiation and reduced bone resorption in the supplemented rats.

Previously, lack of anti-inflammatory cytokines IL-4 and IL-10 has been shown to lead to accelerated bone loss [73]. A previous study has shown that at the completion of chemotherapy treatment for bowel cancer involving Folfox chemotherapy (combination of 5-Fluroruracil, Folic acid and Oxaliplatin), levels of IL-4 and IL-10 were reduced in plasma [74]. In the current study, MTX did not affect mRNA expression of IL-4 and IL-10; and only fish oil supplementation modestly affected IL-4 plasma levels and none of the other supplementary groups had any effects on IL-10 plasma levels. Our current study suggests that MTX chemotherapy, apart from causing an osteoclastogenic and pro-inflammatory condition in the bone, did not significantly lower the expression levels of anti-inflammatory cytokines (IL-4 and IL-10), and fish oil and/or genistein supplementation was not found to affect the levels of these anti-inflammatory cytokines.

Previous studies have shown that omega-3 fatty acid-derived lipid mediators, known as resolvins and protectins, may play an important role in resolving inflammation since they have potent anti-inflammatory properties [75], [76]. Osteoclast mediated bone resorption is often a result of increased inflammation and studies have demonstrated that both resolvins and protectins inhibit inflammation-induced bone resorption and directly modulate osteoclast differentiation and prevent bone resorption, providing a mechanism by which the n-3 PUFAs protect against bone loss [75], [77]. However, it remains to be investigated whether chemotherapy with fish oil in this study would alter the profile or relative abundance of lipid mediators of pro-inflammatory as opposed to anti-inflammatory/proresolving lipid mediators in the bone, bone marrow and in the circulation. It may be possible that these lipid mediators might have promoted resolution of inflammation by suppressing production of inflammatory/pro-osteoclastogenic cytokines and promoted production of anti-inflammatory cytokines in this study. On the other hand, genistein, having an anti-inflammatory property, has been shown to inhibit inflammatory transcription factor nuclear factor kappa B (NF-*κ*B) activation (known to be important for osteoclast formation, function and survival). Genistein inhibits RANKL-induced I-κB degradation and NF-κB nuclear translocation and hence inhibiting differentiation of osteoclasts [28]. In addition, genistein has been shown to directly suppress TNF-α-induced osteoclastogenesis and bone loss via suppression of c-fos expression in osteoclast precursors, and this suppression has been proposed to prevent nuclear accumulation of nuclear factor of activated T cells (NFATc1), a key regulator of osteoclast formation [54]. Further studies are required to investigate the exact mechanisms of action of n3-PUFAs and genistein in attenuating or counteracting MTX chemotherapy-induced inflammatory condition, osteoclast formation and bone resorption during MTX chemotherapy.

In summary, this study examined the effects of dietary supplement with fish oil and/or genistein on long bones in rats subjected to MTX chemotherapy. MTX induces an inflammatory condition, increases osteoclast formation and stimulates adipogenesis at the expense of osteogenesis, thus leading to bone loss. Supplementation with fish oil and/or genistein conserves the bone and prevents MTX chemotherapy-induced bone loss by suppressing osteoclastogenesis and stimulating osteogenesis while concurrently inhibiting adipogenesis in bone marrow. No additive or synergistic protection was observed when fish oil and genistein were administered in combination in this current study, although two previous studies have shown that genistein and fish oil additively induced parameters of bone structure and increased bone mass synergistically in an ovariectomy-induced bone loss model [78], [79]. Despite this, the promising effects of fish oil and genistein, shown in the current study, suggest that their therapeutic potential in preventing MTX chemotherapy-induced bone loss warrants further evaluation. Further studies are required to elucidate the action mechanisms involved and the optimal doses of these supplements required in preventing chemotherapy-induced bone loss, including in models of longer-term chemotherapy or with other/multiple chemotherapy drugs.
